# Public healthcare system utilization for chronic hepatitis C infection in Vietnam

**DOI:** 10.1186/s12879-023-08726-7

**Published:** 2023-11-16

**Authors:** Phuong Nguyen Thi Ngoc, Ngoc Nghiem My, Sabrina Rasheed, Fatema Khatun, Jennifer Van Nuil, Dung Nguyen Thanh, Hung Le Mạnh, Motiur Rahman

**Affiliations:** 1 Wellcome Asia Africa Programme, The Hospital for Tropical Diseases, Oxford University Clinical Research Unit, Ho Chi Minh City, Ho Chi Minh City, Vietnam; 2 The Hospital for Tropical Diseases, Ho Chi Minh City, Vietnam; 3 International Center for Diarrhoeal Disease Research, Bangladesh (icddr,b), Dhaka, Bangladesh; 4 Centre for Tropical Medicine and Global Health, Nuffield Department of Medicine, Oxford University, Oxford, UK

**Keywords:** Gender, Hepatitis C virus, Health insurance, Determinants, HCV treatment, Hospital visits

## Abstract

**Background:**

Healthcare utilization is typically adversely affected when the treatment is expensive and requires multiple visits. We examined the determinants of healthcare-seeking for Hepatitis C virus (HCV) infection which is asymptomatic, chronic, and requires costly treatment in an urban tertiary care referral hospital in Vietnam.

**Methods:**

We conducted a secondary analysis of hospital data for patients attending the Hospital for Tropical Diseases in Ho Chi Minh City, Vietnam between 2017 and 2020 specifically for HCV infection treatment. Poisson regression was used to determine the effect of personal factors (age, sex, comorbidities) and structural factors (health insurance, proximity to the facility, seasonality, year of visit) on the number of hospital visits.

**Results:**

From 2017 to 2020 a total of 22,052 eligible patients sought treatment in the hospital. Among the patients, 50.4% were males and 58.7% were > 50 years of age. The mean number of visits per person was 2.17. In the multivariate analysis compared to 2017, the number of hospital visits increased by 4% in 2018 and then significantly decreased in 2019 and 2020. Visit numbers were significantly lower (6%) among South East region residents compared to those from Central Highlands and for those who lived further away from the hospital. The visit numbers were significantly higher among older age groups (5–11%), those with health insurance (6%), and those with comorbidities (5%) compared to others. Although the number of hospital visits by females was higher (7%) than males in 2017, it significantly decreased in subsequent years.

**Conclusions:**

Our study indicated that there are both structural and individual factors affecting the number of visits for HCV treatment. To meet the global strategy for elimination of HCV, Vietnam Government needs to address the structural and personal barriers to healthcare seeking, with a special focus on women.

## Background

Providing equitable access to health care is at the core of national health systems around the world. However, data points to the fact that disparities in access, especially among the poor and females, remain prevalent [1]. In terms of healthcare utilization, researchers have reported that sex, age, socio-economic status, type of illness (acute or chronic), access to services (cost and proximity), and perceived quality of the service tend to affect an individual’s healthcare utilization [2]. From the patient’s perspective, healthcare-seeking behavior is often influenced by the perceived consequences of the illness, the intensity of symptoms, and trust in the services/health facility [3]. Gender plays an important role in determining the types of healthcare utilized. Women were more likely to receive accessible, less expensive primary healthcare, while men were more likely to receive more expensive specialist inpatient care [4]. In the case of Hepatitis C virus (HCV) infection, many of these barriers are likely to be present as well.

HCV infection is a chronic infection that remains asymptomatic in the majority of patients in the early stage of the infection. For many patients, HCV infection is often diagnosed during screening for other illnesses or during routine health screening [5]. The WHO Western Pacific Region, including Vietnam, bears the highest burden of HCV infection with approximate 14 million chronic infections [5]. Chronic HCV prevalence is higher among men due to higher HCV transmission risk factors including injection drug use and sexual risk behaviors [6–8]. HCV can be treated with direct acting antiretroviral agents (DAAs) and > 90% of the patients are cured with 12 to 24 weeks of treatment [9]. The average cost for a 12 and 24 weeks of DAA treatment in Vietnam is US$2398 and US$ 4284 [10] respectively. In Vietnam, HCV treatment is offered to patients who are HCV RNA positive and HCV genotype determined. In addition, HCV viral load testing (approximate cost 55 US$/test), the key test for enrollment in treatment, is available only in provincial/regional and/or specialized hospitals designated for treatment of HCV infection in Vietnam. HCV treatment requires multiple follow up visits (initial visit, 4 weeks, 12 or 24 weeks after initiation of treatment, and 12 weeks after completion of treatment) and laboratory investigations (full blood count, liver and kidney function test, and HCV viral load assay) during each follow up visit [11] making it difficult for patients in terms of cost of treatment and opportunity costs of multiple visits.

Data related to health care utilization for HCV treatment among adults are limited in Vietnam. Ministry of Health, Vietnam approved the use of DAAs as first line of treatment for HCV in 2017 and provincial hospitals and designated HCV referral hospitals rolled out DAAs the same year. Since then, HCV patients from defined geographic regions were referred to Hospital for Tropical Disease (HTD), Ho Chi Minh City, Vietnam. For HCV care and treatment, it is important that the determinants of visits to healthcare facilities for treatment are assessed to understand the barriers and facilitators to health care utilization. In the current paper, we assessed the determinants of hospital visits for HCV treatment among adults in Vietnam.

## Materials and methods

### Study description and ethical approval

We performed a secondary data analysis of records for patients who attended the hepatitis outpatient department of HTD for HCV care and performed a HCV viral load assay for DAAs treatment enrollment between January 2017 and December 2020. Inclusion criteria included: age ≥ 18 years and residents of central (3 sub-regions with 17 provinces) and southern regions (2 sub-regions with 21 provinces). We excluded patients admitted in the in-patient department or patients with missing geolocation information. The study received ethical approval from the Ethics Review Committee of the Hospital for Tropical Diseases (approval no CS/ND/16/02 date 23/11/2017).

### Setting, patients and data extraction

HTD, is a 650-bed infectious disease hospital, and a designated specialized care provider and referral center for patients with infectious hepatitis from the central and southern Vietnam [10]. In 2015, HTD introduced an electronic record keeping system for the outpatient departments. Data recorded in the system include sociodemographic data including geolocation, as well as clinical, imaging, prescribing, and diagnosis and treatment outcomes for each patient under a unique identification number (ID). The HTD clinical laboratory maintains a separate database of all laboratory investigations conducted on patient samples; laboratory data are stored using a separate laboratory ID linked to the unique patient ID. The laboratory service usage by a patient was identified by linking the hospital ID number with the laboratory database. For this study, the hospital database was screened for the HCV treatment and patients with treatment were further screened for HCV viral load assays conducted during the four-year time period. The hospital records management team extracted sociodemographic and geolocation data (at division, district, and commune levels) for all eligible patients from the database according to the study protocol. All patient data were anonymized by replacing the patient ID (unique ID) with a unique study number before transferring data to the study investigators.

### Data variables

At first visit prior to the HCV treatment initiation at the hospital (HTD) data on age, sex, geolocation (to the commune level), health insurance status, and comorbidities (diabetes, HIV and HBV infection) were collected. The date and times of hospital visits were collected subsequently. The geolocation was extracted from patient address. Geo-location data (at ward level) was collected using Google GIS coordinates and distance was determined by QGIS software. Hospital visit and HCV viral load test date were used to determine the seasonality of the visits. In the central and southern regions, there are two seasons (i.e. rainy season from April to October and winter season from November to March as defined by metrological department of Vietnam). Number of times tested was identified by linking the laboratory database with the patient’s unique hospital ID number.

### Data analysis

The main outcome of interest was the number of hospital visits for HCV treatment. Independent variables included age, sex, area of residence, distance from the HTD and seasonality. As the outcome of interest was number of hospital visits which is a count data, we conducted bi-variate Poison regression to assess the association between independent variables and hospital visits. Then multivariate Poisson regression was used to control the effect of multiple independent variables. Further, we assessed the interaction of gender and other independent variables on the number of hospital visits for HCV treatment. The data was assessed for over dispersion. We have used 5% level of significance as a cut of to determine association between independent and dependant variables. Data analysis was performed using Statistical Package for Social Science (SPSS) software (IBM SPSS Statistics 23, NY USA) and R version 3.6.1.

## Results

From January 1, 2017 to December 31, 2020 of 22,162 patients visited HTD hepatitis outpatient department for HCV treatment and HCV viral load assay. We excluded data for 70 patients from the inpatient department, 40 from the northern region and one patient with missing geolocation data. Finally, data from 22,052 patients were included in the analysis.

Among the study population, 41% visited the HTD in 2017 and there was a steady decline in facility visits with 2020 displaying the lowest proportion of visits for HCV treatment (Table 1). Among study participants 58.7% of patients were over 50 years of age; 50.4% were males; 91.8% were from the Southern region which is where the hospital was located; 72.3% patients did not have any health insurance; and 92.4% patients did not report any comorbidity.

**Table 1:** Characteristics of the population

Characteristics	Total N = 22,052 n (%)
Year	
2017	9033 (41.0)
2018	5600 (25.4)
2019	4527 (20.5)
2020	2892 (13.1)
Age (years)	
18–30	1059 (4.8)
31–40	3791 (17.2)
41–50	4258 (19.3)
> 50	12,944 (58.7)
Gender	
Male	11,110 (50.4)
Female	10,942 (49.6)
Region	
Central	1808 (8.2)
Southern	20,244 (91.8)
Sub-region	
Central Highlands	858 (3.9)
North Central	41 (0.2)
South Central Coast	909 (4.1)
Southeast	9849 (44.7)
Southwest	10,395 (47.1)
Insurance	
Yes	6102 (27.7)
No	15,950 (72.3)
Comorbidities (diabetes, HIV and HBV infection)	
Yes	1681 (7.6)
HIV (n = 17,884)	364 (2.0)
HBV (n = 17,884)	929 (5.1)
Diabetes ( n = 17,884)	158 (0.8)
No	20,371 (92.4)

**Fig. 1:**
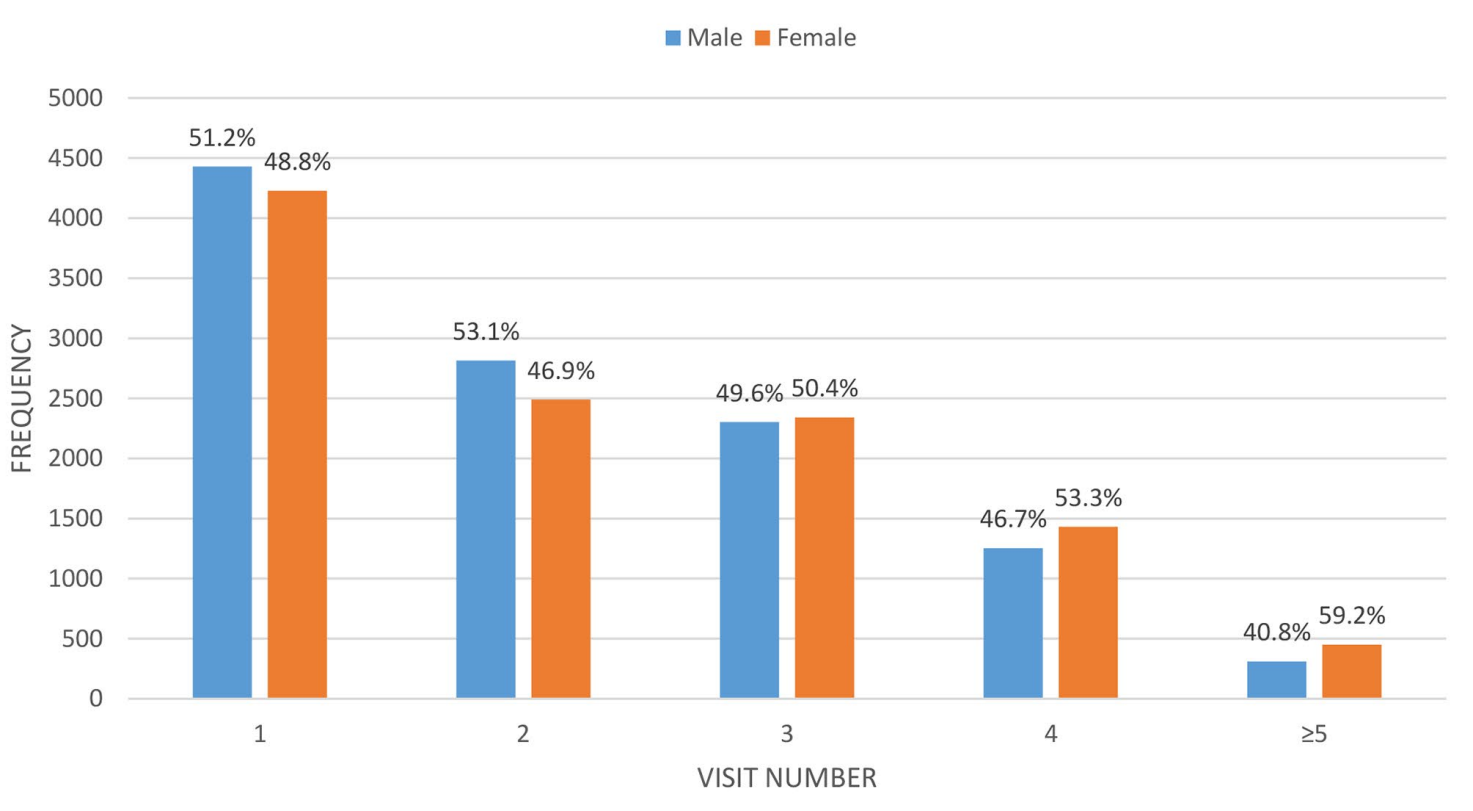
Distribution of visit frequency for HCV treatment at HTD from 2017–2020 by gender

The patients who sought care for HCV visited the hospital multiple times (mean = 2.17). The visit frequency was skewed to the left meaning that the number of people who visited the HTD for care was the highest for the first visits and declined for each subsequent visit (Fig. 1). Given that the standard number of visits required to complete HCV treatment was four, the data indicates that for many patients treatment was not complete.

In the bivariate Poisson regression to assess the association between different independent variables and number of hospital visits for HCV treatment we found that hospital visits in 2017 was significantly higher than in 2019 and 2020 (p < 0.001) (Table 2). Significantly less number of visits were made by patients during rainy season compared to dry season (p = 0.007). Compared to residents of Central highland, only those who lived in Southwest sub-region made significantly higher number of hospital visits for treatment (p = 0.032). Compared to males, female patients visited the hospital significantly greater number of times for treatment (p < 0.001) (Table 2). Older patients were more likely to visit the HTD significantly greater number of times (p < 0.001) compared to those aged 18–30 years. Patients with health insurance coverage visited HTD significantly more times than those without insurance (p < 0.001).

**Table 2:** Association between independent variables and number of hospital visits for HCV treatment

Variable	β	Exp (β)	p-value
**Year**			
2017	Reference		
2018	0.012	1.012	0.290
2019	-0.125	0.883	0.000
2020	-0.381	0.683	0.000
**Season**			
Dry	Reference		
Rainy	-0.025	0.975	0.007
**Sub-region**			
Central Highlands	Reference		
North Central	-0.134	0.874	0.255
South Central Coast	0.035	1.035	0.287
Southeast	0.026	1.026	0.289
Southwest	0.053	1.054	0.032
**Gender**			
Male	Reference		
Female	0.045	1.046	0.000
**Age group**			
18–30 years	Reference		
31–40 years	0.062	1.064	0.012
41–50 years	0.097	1.102	0.000
> 50 years	0.131	1.139	0.000
**Distance to** HTD **(km)**	-0.0001	1.000	0.002
**Insurance**			
No	Reference		
Yes	0.038	1.039	0.000
**Comorbidities (diabetes, HIV and HBV infection)**			
No	Reference		
Yes	-0.0001	1.000	0.996

In the multivariate analysis compared to 2017, in 2018 the number of hospital visits increased by 4% while the number of hospital visits significantly decreased in 2019 and 2020 (Table 3). Compared to residents of Central highlands the number of visits decreased by 6% among the residents of Southeast region. Compared to 18–30 years old patients the number of hospital visits increased by 5% (31–40 age group), 9% (41–50 age group) and 11% (> 50 age group). The number of hospital visits were 7% more among female patients, compared to male patients (Table 3). Compared to those without insurance the number of hospital visits were 6% higher among those with health insurance. Patients who reported comorbidities made 5% more visits to hospital compared to those with no comorbidities. There was a significant inverse relationship between the distance to HTD and the number of hospital visits. When we looked at the interaction of gender with year of hospital visit, we found that compared to males in 2017 the number of hospital visits by females significantly decreased over time.

**Table 3:** Multivariate association between independent variables and number of hospital visits for HCV treatment

Variable	β	Estimated IRR	p-value
Year			
2017	Reference		
2018	0.039	1.040	0.014
2019	-0.086	0.918	0.000
2020	-0.323	0.724	0.000
Sub region			
Central Highlands	Reference		
North Central	0.131	1.140	0.304
South Central Coast	0.037	1.038	0.255
Southeast	-0.059	0.944	0.048
Southwest	-0.013	0.988	0.619
Age group			
18–30 years	Reference		
31–40 years	0.053	1.055	0.031
41–50 years	0.085	1.089	0.000
> 50 years	0.103	1.109	0.000
Gender			
Male	Reference		
Female	0.065	1.067	0.000
Insurance			
No	Reference		
Yes	0.026	1.027	0.012
Comorbidities (diabetes, HIV and HBV infection)			
No	Reference		
Yes	0.051	1.051	0.003
Distance to HTD (km)	-0.0003	1.000	0.000
Year X gender			
2017, Male	Reference		
2018, Female	-0.046	0.956	0.039
2019, Female	-0.067	0.937	0.007
2020, Female	-0.116	0.891	0.000

IRR: incidence rate ratio

## Discussion

This paper describes factors associated with number of visits to one tertiary public hospital(HTD) that serves as the referral clinic for two thirds of Vietnam for HCV treatment. Our analysis indicated that there are both structural and individual factors that affect healthcare seeking for HCV treatment, which requires multiple visits to the hospital. In previous studies researchers reported the prevalence and risk factors for HCV infection in Vietnam [12–14]. However, in this paper we assessed the factors associated with the continuity of care for the treatment of HCV infection.

In our study, structural factors such as year of starting the treatment, distance from health facility, and having any health insurance was associated with number of hospital visits for HCV treatment. After the DAA treatment was made available in public hospitals in 2017, the number of hospital visits for HCV treatment was high, a level that continued in 2018. It is possible that for the first two years the high patient flow was due to fulfillment of unmet treatment need as low-cost treatment was made available in the public hospital [10]. The subsequent decline in visit frequency may be due to a smaller patient pool as most diagnosed HCV patients may have been treated within the first two years. The number of visits made in 2020 was the lowest probably due to the COVID-19 restrictions which have been shown to reduce health care utilization in many countries around the world [15]. We found that the distance to health facilities was a significant predictor of number of visits for treatment. As distance increased the number of facility visits decreased. In Vietnam and other low-and-middle income countries distance to health facilities have shown to be a significant barrier to health care utilization [16–18]. Distance adds transportation cost and opportunity cost to care seeking which could be difficult for many [19, 20]. In our study having health insurance was associated with higher number of visits per patient. Although at first visit those with no insurance were more likely to initiate treatment, for all the subsequent visits those with health insurance were more likely to seek treatment. Health insurance has shown to reduce out of pocket expenditures for treatment and could lead to increased health care utilization in other low and middle income countries [21]. It is important to note that the number of patients who visited the hospital for HCV treatment was the highest for the first visit and declined subsequently. This could be either because patients could not afford to seek care and or conducting the HCV viral load test despite the availability of low-cost treatment in the hospital or they went to private sector for care seeking.

The individual factors associated with number of hospital visits for HCV treatment include age, existing comorbidities and gender. The number of hospital visits for HCV treatment increased with age. The number of HCV patients in 18–30 age group was only 4.8% which may mean that the disease is identified at a later age when health check-up takes place for other health conditions [22]. Among the patients 0.8%, 2.0% and 5.1% had diabetes, HIV and HBV infection respectively. We found that those with co-morbidities (diabetes, HIV and HBV infection) tend to make more visits for HCV treatment than others. It is possible that when a patient seeks treatment for a health condition, chances of other health conditions such as HCV are more likely to be detected and dealt with. It is also possible that those who frequent hospitals for different health conditions could be at higher risk of HCV transmission as in previous studies researchers reported a 2.1 to 3.7 times higher risk of acquiring HCV among those who frequented the hospitals and had blood transfusion or other invasive procedures [23, 24]. Women tend to visit the health facility more frequently for HCV treatment than men. However, when we examined the interaction of gender and year of first visit we found that the number of visits by women was significantly more in only 2017 and in the subsequent years, women made significantly less number of visits than men. This finding indicates that females face barriers in healthcare utilization and the barrier increases when multiple visits to hospital are required for HCV treatment. Similar findings about inequities faced by women have been seen in Vietnam and other LMICs for other health conditions such as tuberculosis that require long term treatment [25].

In 2016, the WHO the launched a global strategy to eliminate HCV as public health threat by 2030 [26]. A key priority in these recommendations is to ensure universal access to the DAAs [27] although it is acknowledged that people in LMICs might not be able to afford the treatment recommended. To adhere to the global strategy, Vietnam has made efforts to make DAA available in public hospitals at low cost to addressed some of the unmet need for treatment. Despite the efforts, for a large number of those who were patients of HCV did not complete treatment as indicated by less than 4 hospital visits required for treatment completion. It is important therefore, that the existing structural and individual barriers to health care seeking that still exist, are addressed to ensure early diagnosis and treatment completion.

### Limitations

Our study has several limitations; we analyzed the care seeking behaviors at one public tertiary care facility, which might be different from private or primary care facility. We lack data on first diagnosis of HCV infection and therefore, could not estimate delays in HCV care seeking. We did not have any information about care seeking for HCV in other health facilities for a patient which could lead to underestimation of rates of treatment completion. Further, we did not have data on the socioeconomic status (household income or expenditure) of the patients that would have enriched the analysis.

## Conclusions

Our study indicated that there are both structural and individual factors affecting the number of visits for HCV treatment. The structural factors included year of treatment, distance from health facility and having health insurance and individual factors included age, existing comorbidities and gender. To meet the global strategy for elimination of HCV it is important that the barriers to healthcare seeking are addressed adequately.

## Data Availability

All data is available through Oxford University Clinical Research Unit data sharing policy. Contact for data: DAC@oucru.org.
